# Associations of screen-based sedentary activities with all cause dementia, Alzheimer’s disease, vascular dementia: a longitudinal study based on 462,524 participants from the UK Biobank

**DOI:** 10.1186/s12889-023-17050-3

**Published:** 2023-11-02

**Authors:** Shiqi Yuan, Wanyue Li, Yitong Ling, Xiaxuan Huang, Aozi Feng, Shanyuan Tan, Ningxia He, Li Li, Shuna Li, Anding Xu, Jun Lyu

**Affiliations:** 1 Department of Neurology, Guangdong Province, The First Affiliated Hospital of Jinan University, No.613, Huangpu Road West, Guangzhou, 510630 China; 2 Department of Rehabilitation, Guangdong Province, The First Affiliated Hospital of Jinan University, No.613, Huangpu Road West, Guangzhou, 510630 China; 3 Department of Clinical Research, Guangdong Province, The First Affiliated Hospital of Jinan University, No.613, Huangpu Road West, Guangzhou, 510630 China; 4 Guangdong Provincial Key Laboratory of Traditional Chinese Medicine Informatization, Guangzhou, 510630 Guangdong China

**Keywords:** TV viewing, Genetic susceptibility, Brain structure, Dementia, Bidirectional Mendelian randomization, UK Biobank

## Abstract

**Background:**

Current drug treatments for dementia aren't effective. Studying gene-environment interactions can help develop personalized early intervention strategies for Alzheimer's disease (AD). However, no studies have examined the relationship between screen-based sedentary activities and genetic susceptibility to AD risk, and further understanding of the causal relationship is also crucial.

**Methods:**

This study included 462,524 participants from the UK Biobank with a follow-up of 13.6 years. Participants' screen-based sedentary activities time was categorized into three groups based on recorded time: ≥ 4 h/day, 2–3 h/day, and ≤ 1 h/day. Cox proportional risk models were used to analyze the association between computer use/TV viewing groups and the risk of all-cause dementia, AD and vascular dementia (VD). Generalized linear model (GLM) were used to examine the relationship between screen-based sedentary activities and brain structure. Bidirectional Mendelian randomization (MR) was used to validate the causal relationship between TV viewing and AD.

**Results:**

Compared to TV viewing ≤ 1 h/day, 1)TV viewing 2–3 h/day was correlated with a higher risk of all-cause dementia (HR = 1.09, 95% CI:1.01–1.18, *P* < 0.05), and TV viewing ≥ 4 h/day was associated with a higher risk of all-cause dementia (HR = 1.29, 95% CI: 1.19–1.40, *P* < 0.001), AD (HR = 1.25, 95% CI:1.1–1.42, *P* < 0.001), and VD (HR = 1.24, 95% CI: 1.04–1.49, *P* < 0.05); 2) TV viewing ≥ 4 h/day was correlated with a higher AD risk at intermediate (HR = 1.34, 95% CI: 1.03–1.75, *P* < 0.001) and high AD genetic risk score (AD-GRS) (HR = 2.18, 95% CI: 1.65–2.87, *P* < 0.001);3) TV viewing 2–3 h/day [β = (-94.8), 95% CI: (-37.9) -(-151.7), *P* < 0.01] and TV viewing ≥ 4 h/day [β = (-92.94), 95% CI: (-17.42) -(-168.46), *P* < 0.05] were correlated with a less hippocampus volume. In addition, a causal effect of TV viewing times was observed on AD analyzed using MR Egger (OR = 5.618, 95%CI:1.502–21.013, *P* < 0.05).

**Conclusions:**

There was a causal effect between TV viewing time and AD analyzed using bidirectional MR, and more TV viewing time exposure was correlated with a higher AD risk. Therefore, it is recommended that people with intermediate and high AD-GRS should control their TV viewing time to be less than 4 h/ day or even less than 1 h/day.

**Supplementary Information:**

The online version contains supplementary material available at 10.1186/s12889-023-17050-3.

## Background

With the aging of population, dementia has become a major public concern worldwide and is expected to affect 1.5 million people by 2050, placing an enormous burden on society [1, 2]. Current drug treatments for dementia, especially Alzheimer's disease (AD), have not yielded satisfactory results [3]. More and more risk factors for dementia have been identified by evidence-based medicine, which has promoted the development of dementia prevention strategies [4].

AD is attributed to the combination of genetic and environmental factors [5]. Study of gene-environment interactions contributing to AD development can help develop personalized early AD intervention strategies to dramatically decrease AD incidence worldwide [6]. Today's people keep sedentary status during nearly two-thirds of their leisure time with the most popular activities including watching TV and using computers [7]. Studies showed that excessive TV viewing is associated with cognitive decline [8, 9]. However, there is no study on the relationship between screen-based sedentary activity and genetic susceptibility to AD risk.

Magnetic resonance imaging (MRI) can be applied to detect different types of structural and functional abnormalities in dementia [10]. Research is needed to study the relationship between screen-based sedentary activity and brain structure.

Understanding the causal relationship of screen-based sedentary activities and AD is critical for prevention of AD. Traditional observational epidemiological studies have many limitations in investigation of disease etiology and inferencing causality (e.g., reverse causal associations and potential confounding factors) [11]. Mendelian Randomization (MR) research design following the Mendelian inheritance law of "parental alleles are randomly assigned to offspring", and MR based on genetic variations are very helpful for overcoming these limitations [12, 13]. In our study, to obtain a more reliable and definitive causal relationship of screen-based sedentary activities and AD, bidirectional MR was used to further validate the findings of our observational study.

Based on the UK Biobank, the aim of this study was to investigate the relationship of screen-based sedentary activity (computer use and TV viewing) and vascular dementia (VD), all-cause dementia, and AD. In addition, we also explored the correlation between genetic susceptibility and screen-based sedentary activity and AD risk. Moreover, we investigated the association of screen-based sedentary activity with brain structure. Bidirectional MR was then utilized to validate the causal association of TV viewing and AD.

## Methods

### Study population

UK Biobank (UKB) is a frequently used prospective cohort for risk factor studies on major diseases in and old and middle-aged adults. More than half a million women and men (40–69 years old) were recruited between 2006 and 2010, and their health status was tracked over time. Since its inception in 2006, UKB has collected blood, urine, and saliva samples, genetic and imaging data, and demographic, health, lifestyle, social and economic information from more than 500,000 UK participants [14]. The UKB study was approved by the Northwest Multicenter Research Ethics Committee (11/NW/0382), and all participants signed written informed consent forms [15, 16]. Our study obtained the UK Biobank license with application ID of 76,636.

The exclusion criteria were as follows: previous all-cause dementia, AD or vascular dementia (*n* = 253); participants who lacked complete baseline data records, genetic data, and exposure data (*n* = 39,617). Eventually, among the 502,394 original participants, 462,524 participants were included in this study.

**Fig. 1:**
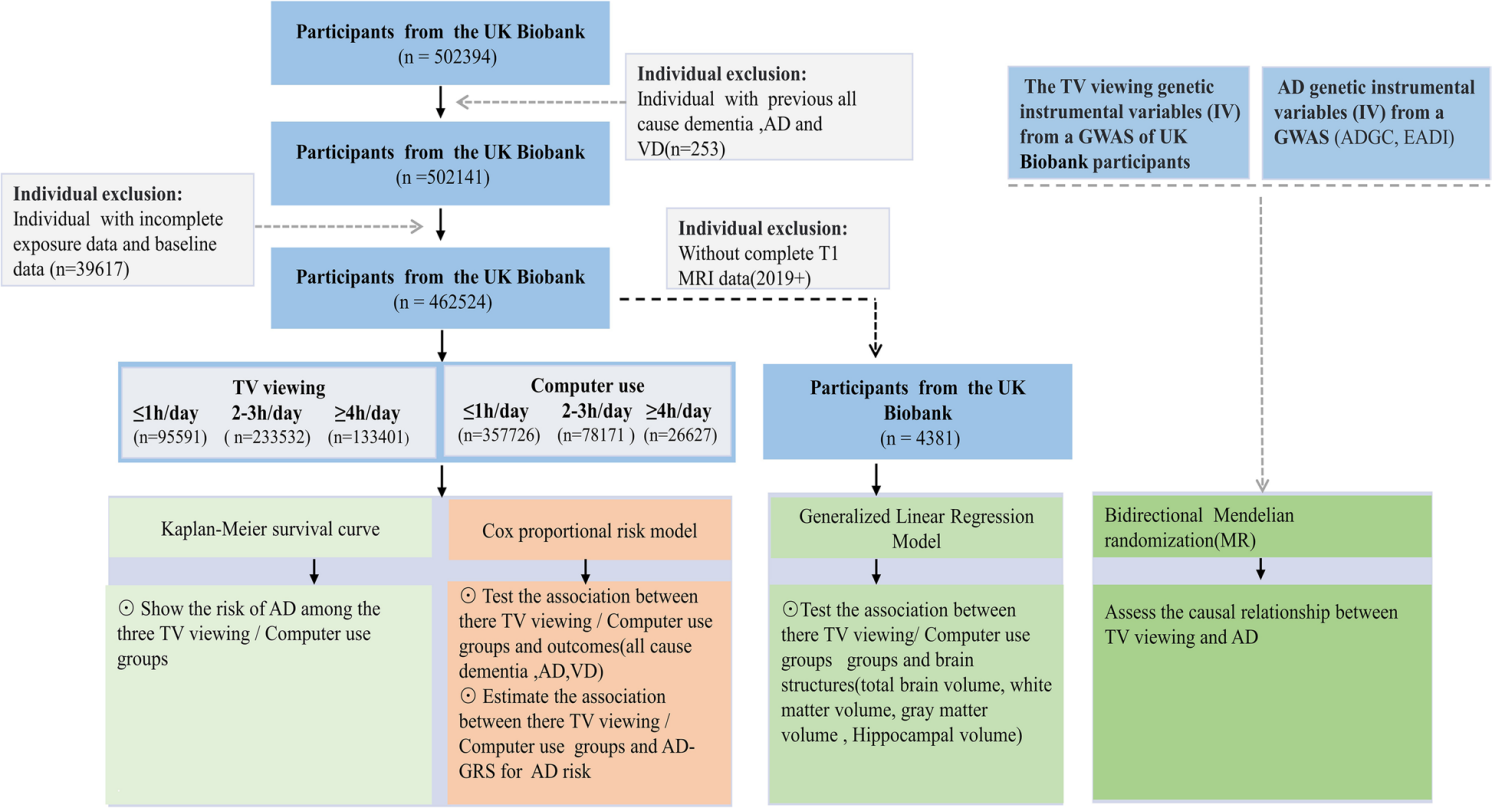
The flow chart of the study. VD, vascular dementia; AD, Alzheimer's disease; MRI, Magnetic resonance imaging

In addition, as changes in brain structure are a long-term process, 4,831 of the 462,524 participants had complete MRI data after 2019 (Instance 3: imaging visit,2019 +) and were selected for structural brain analysis. All participants had complete case analysis. Figure 1 shows the flow chart of the study.

### Exposures

Exposure factors in our study were based on screen-based sedentary activities, including television viewing and computer use [7]. All the participants finished a questionnaire including questions such as computer use in leisure time and TV watching. Based on the recorded time for screen-based sedentary activities (Filed ID: 1080, 1070), three categories of TV viewing and computer use were generated: ≥ 4 h/day, 2–3 h/day and ≤ 1 h/day [17].

### Confounders

A series of sociodemographic, behavioral, and cardiovascular disease confounding factors associated with dementia were selected based on the literature [2, 18]. The confounders included age, sex, body mass index (BMI), education level, Townsend deprivation index (TDI), ethnicity, myocardial infarction (MI), alcohol use, hypertension, smoking, diabetes, and stroke. The confounder variables were obtained from UKB database registration records and hospital diagnosis records.

### Outcomes

The primary outcome included VD, all-cause dementia, and AD. The outcome events were determined according to the UKB database algorithmically-defined outcomes and the records of the outcome of the hospital diagnosis (Field ID :42018,130840,130842,42020,130836,42022,130838,42024). Follow-up time was the time when the outcome event occurred, the time of registered death, or the time when the final outcome occurred (November 13, 2021), whichever occurred first.

### Brain MRI

The scanner used for brain MRI data capture is a standard Siemens Skyra 3 T running VD13A SP4, with a standard Siemens 32-channel RF receive head coil, The processing of brain MRI data has been reported previously [19]. T1-weighted imaging, a high-resolution structural MRI technique producing a strong contrast between white and gray matters, was used for test of brain anatomy [20], and the brain (gray matter + white matter) volume (mm^3^) (normalized for head size), gray matter volume, white matter volume, and hippocampus volume were selected and analyzed (Field ID : 25009,25007, 25005, 25019, 25020).

### Assessment of genetic susceptibility to Alzheimer's disease

Polygenic risk scores (PRS) are often used for genetic risk assessment for AD. Previous study showed the genotyping of UKB participants and input procedures for quality control of genetic data [21]. In this study, to reduce the high genetic risk score of false positives, the newly discovered UKB database single nucleotide polymorphisms (SNPs) were not involved in our score. The 29 SNP sites that were highly correlated with AD were selected from previous Genome-wide association study (GWAS) [22–24]. For each participant, the AD genetic risk score (AD-GRS) was obtained according to the SNPs and the corresponding weight (beta coefficient) as previously described [25]. The beta coefficient and SNPs are shown in Supplemental Table S1. The participants were assigned with low (1 quintile), intermediate (2–4 quartiles), or high (5 quintiles) AD-GRS.

### Bidirectional MR analysis

In bidirectional MR analysis, genetic variation (SNPs) was utilized as an instrumental Variable (IV), and outcome IV and exposure IV were utilized to study the potential causal association between “exposure” and “outcome” [26, 27]. To avoid false positive results, the selected SNPs were utilized to study the potential causal association in the separate GWAS [28]. All genetic variants (SNPs) with time spent in watching television (TV) or AD were selected at *P* < 10^–8^ from the corresponding GWAS. SNPs were clumped for independence with *r*^2^ > 0.001 to remove IVs with linkage disequilibrium [6]. Five methods, including MR Egger Weighted mode, Weighted median, Simple mode, and Inverse variance weighted (IVW) were utilized for MR analysis after data pretreatment to keep SNPs effect alleles and effect sizes uniform ("harmonise_data" in the TwoSampleMR package) [29].

In our study, the AD genetic IV data were acquired from a GWAS of UKB participants with an ID of ieu-b-2, and the time spent in watching TV IV was also acquired from a GWAS with an ID of ukb-b-5192.

First, we investigated when TV viewing was used as an exposure factor whether there was a causal association with AD (outcome) risk. The time spent in watching TV IVs were obtained from a GWAS of UKB participants (Supplemental Table S2). After extracting IV information (Supplemental Table S3) that exposed (the time spent in watching TV) to the outcome (AD), the exposure IVs were matched to the effect allele of outcome IVs (Supplemental Table S4), and eventually 100 SNPs were used as IVs for MR analysis using five methods.

Then, we investigated when AD was used as an exposure factor whether there was a causal association with TV viewing time (outcome). The AD IVs were obtained from ADGC and EADI (Supplemental Table S5). After extracting IV information (Supplemental Table S6) that exposed (AD) to the outcome (the time spent in watching TV), the exposure IVs were matched to the effect allele of outcome IVs (Supplemental Table S7), and eventually 18 SNPs were used as IVs for MR analysis using five methods.

Finally, to obtain reliable MR results, sensitivity analysis of MR results was performed (Supplemental Figure S1- S6), including: 1) heterogeneity test, which tests for differences among IVs; 2) horizontal pleiotropy, which indicates whether there are confounding factors in the study; 3) leave-one-out analysis: the SNPs are removed one by one to determine whether a SNP has significantly changed the results. Through MR Sensitivity test, we selected the most appropriate MR analysis method [30]. IVW corresponds to a weighted regression of the effect of exposure on the outcome with a zero-intercept limit. Due to this limitation, the results may be biased if the instrument SNPS shows horizontal pleiotropy. This may be due to the effect of causal pathways other than exposure on the results [31]. In addition, Weighted Median uses the majority of genetic variants of SNPS to determine the potential causal relationship. The estimate of the MR Egger method is known to be relatively robust for the existence of poly-efficacious [32]. Therefore, MR-Egger regression estimation is used in the case of the existence of pleiotropy. If there is no pleiotropy, the IVW estimate is preferred. When there is only heterogeneity and no pleiotropy, the Weighted Median results are preferred [29, 33].

Two Sample MR Software packages were utilized for MR Analysis and R software was utilized for all statistical analysis.

## Statistical analyses

Baseline features are grouped by TV viewing and Computer use. Numerical variable data are represented by mean ± median (quartile) (non-normal distribution) or standard deviation (normal distribution). Classification variables are represented by the sample size and proportion of each group.

The Kaplan–Meier (K-M) curves were utilized to evaluate the cumulative risk of Computer use/TV viewing and different outcomes (all-cause dementia, AD and VD), and TV viewing and computer use time was divided into three groups: ≥ 4 h/day, 2–3 h/day, and ≤ 1 h/day.

The Cox proportional risk models were applied to investigate the correlation of computer use/ TV viewing groups and all cause dementia, AD, and VD; model 1 was unadjusted; model 2 was adjusted for sex and age; Model 3 was adjusted for education level, ethnicity, sex, age, alcohol use, BMI, TDI, smoking, MI, diabetes, stroke, and hypertension. In addition, we also performed subgroup analyses by sex (Supplemental Figure S7).

The Cox proportional risk models were also used to investigate the association between Computer use/TV viewing, AD-GRS and AD risk. We examined the effect of the interaction of computer use/TV viewing with the AD-GRS on AD risk, and performed a likelihood ratio test to generate an overall interaction test result. The model was adjusted for education level, ethnicity, sex, age, alcohol use, BMI, TDI, smoking, MI, diabetes, stroke, hypertension and AD-GRS. In addition, to further investigate the association between gene-environment combinations and AD risk, participants were categorized into nine groups based on different combinations of AD-GRS (low, intermediate, and high) and TV viewing/Computer use time. We selected the less viewing/Computer use time combined with low AD-GRS as the reference group to examine the association of the other groups with AD risk. The model was adjusted for education level, ethnicity, sex, age, alcohol use, BMI, TDI, smoking, MI, diabetes, stroke, and hypertension.

The generalized linear models (GLM) were applied to investigate the correlation between screen-based sedentary activities and white matter volume, hippocampus volume, gray matter volume, and white matter + gray matter volume. The model was adjusted for ethnicity, sex, age, alcohol use, education level, BMI, TDI, smoking, MI, diabetes, stroke, and hypertension. *P*-value corrected by Benjaminiand and Hochberg (BH) method.

R software was used for statistical analyses, and *P* value < 0.05 was considered statistically significant.

## Results

### Baseline characteristics

Totally our study had 462,524 enrolled UKB participants (median follow-up time: 13.6 years; median age: 58 years; sex: 54.2% male and 45.8% female). There were 6650 new cases of all-cause dementia (1.4%), 2776 new cases of AD (0.6%), and 1514 new cases of VD (0.3%) during follow-up (Table 1**)**.

**Table 1:** Baseline characteristics of participants in three categories of TV viewing and computer use

Characteristics	All	TV viewing			Computer use		
		** ≤ 1 h/day (*n* = 95591)**	**2–3 h/day (*n* = 233532)**	** ≥ 4 h/day (*n* = 133401)**	** ≤ 1 h/day (*n* = 357726)**	**2–3 h/day (*n* = 78171)**	** ≥ 4 h/day (*n* = 26627)**
**Age (Median, IQR)**	58 (50,63)	54 (48,61)	57 (50,63)	61 (54,65)	58 (50,63)	58 (50,63)	55 (48,61)
**TDI (Median, IQR)**	-2.2 (-3.7,0.4)	-2.1(-3.7,0.5)	-2.4 (-3.8,0)	-1.9 (-3.5,1.1)	-2.2(-3.7,0.3)	-2.1 (-3.6,0.7)	-1.7(-3.5,1.3)
**BMI (Median, IQR)**	26.7(24.1,29.1	25.3(23,28.1)	26.6(24.1,29.6)	27.9(25.2,31.3)	26.5(24,29.6)	27.4(24.7,30.6)	27.6(24.8,31)
**Sex (n, %)**							
**Female**	250879 (54.2)	52813 (55.2)	126113 (54)	71953 (53.9)	207076(57.9)	33175 (42.4)	10628 (39.9)
**Male**	211645 (45.8)	42778 (44.8)	107419 (46)	61448 (46.1)	150650(42.1)	44996 (57.6)	15999 (60.1)
**Ethnicity (n, %)**							
**White people**	422157 (91.3)	83775 (87.6)	213922 (91.6)	124460 (93.3)	329378(92.1)	69734 (89.2)	23045 (86.5)
**Mixed people**	16671 (3.6)	3992 (4.2)	8402 (3.6)	4277 (3.2)	12436 (3.5)	2977 (3.8)	1258 (4.7)
**Other people**	23696 (5.1)	7824 (8.2)	11208 (4.8)	4664 (3.5)	15912 (4.4)	5460 (7)	2324 (8.7)
**Education (n, %)**							
**College/University**	153203 (33.1)	52086 (54.5)	79174 (33.9)	21943 (16.4)	108676(30.4)	32374 (41.4)	12153 (45.6)
**Other**	309321(66.9)	43505 (45.5)	154358 (66.1)	111458 (83.6)	249050(69.6)	45797 (58.6)	14474 (54.4)
**Smoking (n, %)**							
**Never**	253414 (54.8)	57805 (60.5)	131176 (56.2)	64433 (48.3)	199695(55.8)	40,201 (51.4)	13518 (50.8)
**Previous**	161005 (34.8)	29797 (31.2)	80251 (34.4)	50957 (38.2)	121456 (34)	29979 (38.4)	9570 (35.9)
**Current**	48105 (10.4)	7989 (8.4)	22105 (9.5)	18011 (13.5)	36575 (10.2)	7991 (10.2)	3539 (13.3)
**Alcohol (n, %)**							
**Never**	19089 (4.1)	4430 (4.6)	8719 (3.7)	5940 (4.5)	14975 (4.2)	2924 (3.7)	1190 (4.5)
**Previous**	16095 (3.5)	3199 (3.3)	6930 (3)	5966 (4.5)	11999 (3.4)	2963 (3.8)	1133 (4.3)
**Current**	427340 (92.4)	87962 (92)	217883 (93.3)	121,495 (91.1)	330752(92.5)	72284 (92.5)	24304 (91.3)
**MI (n, %)**							
**No**	437877 (94.7)	92393 (96.7)	222405 (95.2)	123,079 (92.3)	339420(94.9)	73,433 (93.9)	25024 (94)
**Yes**	24647 (5.3)	3198 (3.3)	11127 (4.8)	10,322 (7.7)	18306 (5.1)	4738 (6.1)	1603 (6)
**Stroke (n, %)**							
**No**	446621 (96.6)	93395 (97.7)	226408 (96.9)	126818 (95.1)	345697(96.6)	75267 (96.3)	25657 (96.4)
**Yes**	15903 (3.4)	2196 (2.3)	7124 (3.1)	6583 (4.9)	12029 (3.4)	2904 (3.7)	970 (3.6)
**Diabetes (n, %)**							
**No**	423209 (91.5)	90858 (95)	216268 (92.6)	116083 (87)	329507(92.1)	70052 (89.6)	23650 (88.8)
**Yes**	39315 (8.5)	4733 (5)	17264 (7.4)	17318 (13)	28219 (7.9)	8119 (10.4)	2977 (11.2)
**Hypertension (n, %)**							
**No**	285914 (61.8)	68971 (72.2)	148194 (63.5)	68749 (51.5)	223768(62.6)	46037 (58.9)	16109 (60.5)
**Yes**	176610 (38.2)	26620 (27.8)	85338 (36.5)	64652 (48.5)	133958(37.4)	32134 (41.1)	10518 (39.5)
**All cause dementia (yes, n, %)**	6650 (1.4)	831 (0.9)	2878 (1.2)	2941 (2.2)	5229 (1.5)	1108 (1.4)	313 (1.2)
**AD (yes, n, %)**	2776 (0.6)	347 (0.4)	1243 (0.5)	1186 (0.9)	2237 (0.6)	424 (0.5)	115 (0.4)
**VD (yes, n, %)**	1514 (0.3)	165 (0.2)	640 (0.3)	709 (0.5)	1179 (0.3)	251 (0.3)	84 (0.3)

### The Kaplan–Meier (K-M) curve

**Fig. 2:**
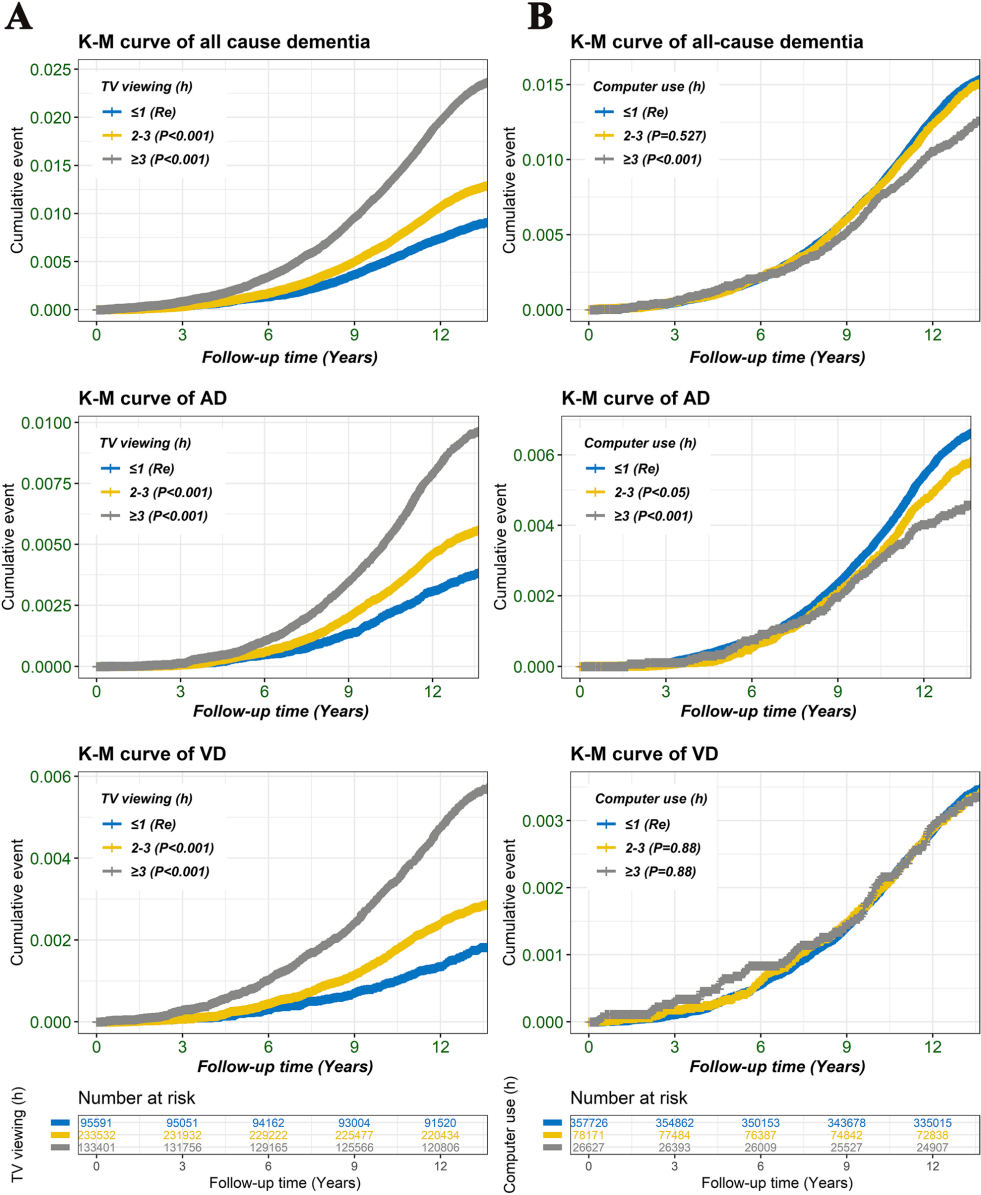
The Kaplan–Meier (K-M) curves were used to assess the cumulative risk between Computer use/TV viewing groups and different outcomes (VD, AD and all-cause dementia). TV viewing and computer use time was divided into three groups: ≥ 4 h/day, 2–3 h/day, and ≤ 1 h/day

The K-M curves were utilized to evaluate the cumulative risk of Computer use/TV viewing and different outcomes (all-cause dementia, AD and VD). As shown in Fig. 2, compared to the group with TV viewing ≤ 1 h/day, the groups with TV viewing 2–3 h/day or ≥ 4 h/day exhibited a higher risk for VD, all-cause dementia and AD. In contrast, the group with computer use ≥ 4 h/day had a lower risk for AD and all-cause dementia compared to the group with computer use ≤ 1 h/day.

### Hazard ratio (HR) estimation for VD, AD and all-cause dementia

The Cox proportional risk models were applied for estimation of the correlation if the computer use/TV viewing groups and outcomes (VD, AD and all cause dementia). As shown in Table 2, after multivariate model adjustment, compared to the group with TV viewing ≤ 1 h/day, the group with TV viewing of 2–3 h/day was correlated with a higher risk for all-cause dementia (HR = 1.09, 95% CI:1.01–1.18, *P* < 0.05), and the group with TV viewing ≥ 4 h/day was associated with a higher risk for AD (HR = 1.25, 95% CI:1.1–1.42, *P* < 0.001), all-cause dementia (HR = 1.29, 95% CI: 1.19–1.40, *P* < 0.001), and VD (HR = 1.24, 95% CI: 1.04–1.49, *P* < 0.05). In addition, after multivariate model adjustment, no obvious correlation was observed between Computer use and VD, AD and all cause dementia (*P* > 0.05). In addition, subgroup analyses by sex showed similar results (Supplemental Figure S7).

**Table 2:** The correlation between TV viewing/Computer use groups and outcomes (VD, all cause dementia, and AD)

Group	All cause dementia		AD		VD	
	**HR (95%CI)**	***P***	**HR (95%CI)**	***P***	**HR (95%CI)**	***P***
**TV viewing (h)**						
**Model 1**						
** ≤ 1**	Re		Re		Re	
**2–3**	1.43(1.32–1.55)	< 0.001	1.48(1.31–1.67)	< 0.001	1.60(1.35–1.90)	< 0.001
** ≥ 4**	2.64(2.44–2.85)	< 0.001	2.54(2.26–2.87)	< 0.001	3.19(2.69–3.78)	< 0.001
**Model 2**						
** ≤ 1**	Re		Re		Re	
**2–3**	1.13(1.05–1.22)	< 0.01	1.15(1.02–1.30)	< 0.05	1.24(1.05–1.47)	< 0.001
** ≥ 4**	1.52(1.41–1.64)	< 0.001	1.41(1.25–1.59)	< 0.001	1.77(1.49–2.09)	< 0.001
**Model 3**						
** ≤ 1**	Re		Re		Re	
**2–3**	1.09(1.01–1.18)	< 0.05	1.12(0.99–1.26)	0.08	1.12(0.94–1.33)	0.202
** ≥ 4**	1.29(1.19–1.40)	< 0.001	1.25(1.1–1.42)	< 0.001	1.24(1.04–1.49)	< 0.05
**Computer use (h)**						
**Model 1**						
** ≤ 1**	Re		Re		Re	
**2–3**	0.98(0.92–0.529)	0.53	0.88(0.79–0.97)	< 0.05	0.98(0.86–1.130)	0.82
** ≥ 4**	0.81(0.81–0.91)	< 0.001	0.7(0.58–0.84)	< 0.001	0.97(0.77–1.2)	0.76
**Model 2**						
** ≤ 1**	Re		Re		Re	
**2–3**	0.92(0.86–0.98)	< 0.01	0.84(0.76–0.94)	< 0.001	0.88(0.77–1.01)	0.07
** ≥ 4**	1.02(0.91–1.15)	0.71	0.93(0.77–1.12)	0.44	1.18(0.94–1.47)	0.15
**Model 3**						
** ≤ 1**	Re		Re		Re	
**2–3**	0.96(0.90–1.02)	0.21	0.9(0.81–1.00)	0.051	0.93(0.81–1.07)	0.31
** ≥ 4**	1(0.89–1.12)	0.99	0.96(0.79–1.16)	0.65	1.11(0.88–1.39)	0.37

The Cox proportional risk models were applied to evaluate the HR of VD, AD, and all cause dementia. Model 1 was unadjusted; Model 2 was adjusted for sex and age; Model 3 was adjusted for education level, ethnicity, sex, age, alcohol use, BMI, TDI, smoking, MI, diabetes, stroke, and hypertension. *AD* Alzheimer's disease, *VD* vascular dementia, *TDI* Townsend deprivation index,* MI *myocardial infarction, *BMI* body mass index, *RE* reference

### Joint correlation between TV viewing/Computer use and AD-GRS for AD risk

**Fig. 3:**
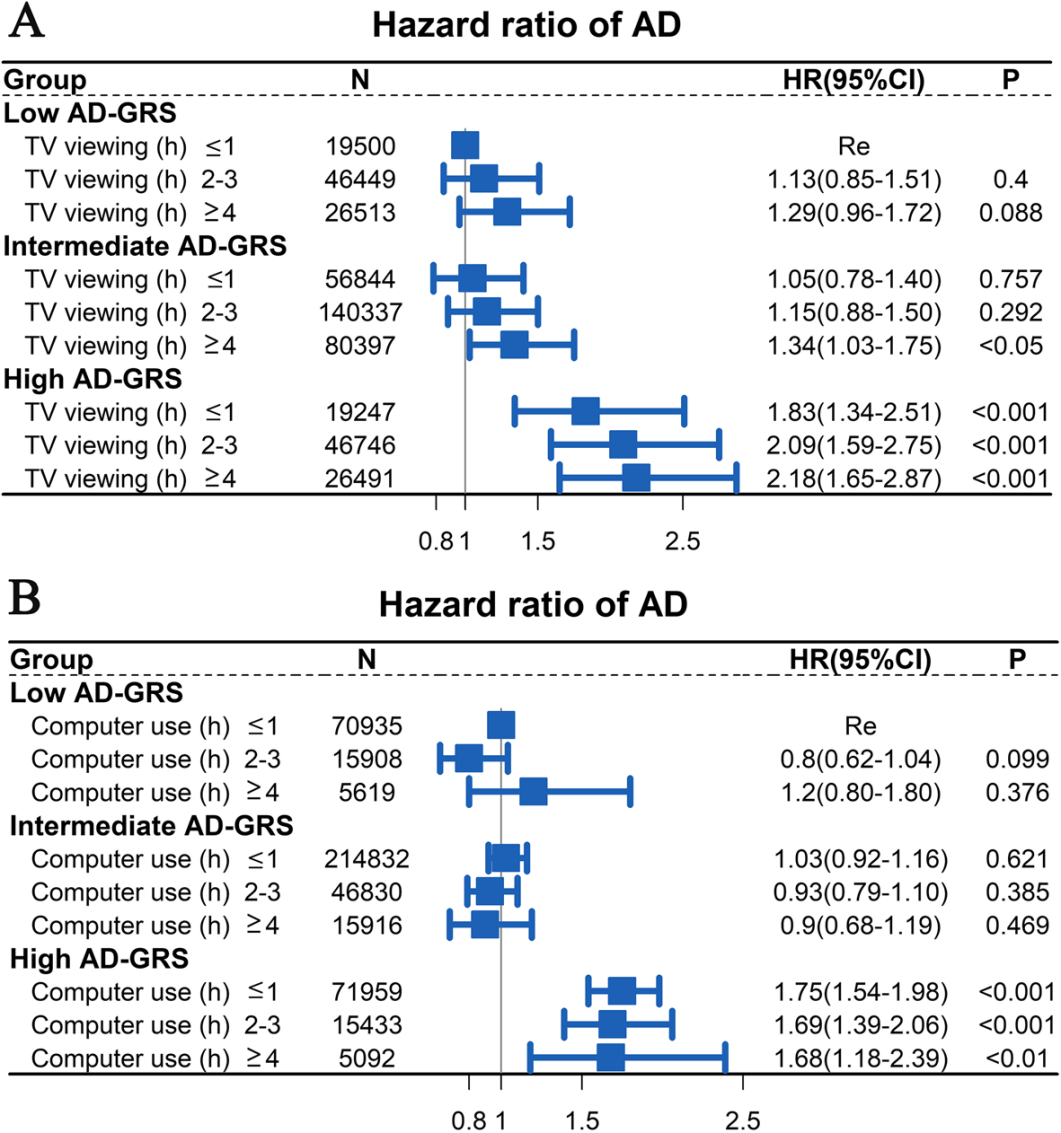
The Cox proportional risk models were applied to investigate the relationship between TV viewing (Fig. 3A)/computer use (Fig. 3B) groups, AD-GRS and AD risk. The less viewing/Computer use time combined with low AD-GRS as the reference group. The model was adjusted for ethnicity, sex, education level, age, alcohol use, BMI, TDI, smoking, MI, diabetes, stroke, and hypertension

The Cox proportional risk models were applied for estimation of the correlation of Computer use/TV viewing time, AD-GRS and AD risk. No significant correlation was found between TV viewing/Computer use time and AG-GRS on AD risk (*P* > 0.05). As shown in Fig. 3, as AD-GRS increased, more AD-GRS participants were associated with a higher AD risk (*P* < 0.001). After the model has been adjusted for multiple variables. As shown in Fig. 3A, an increasing trend was observed in HR with higher AD-GRS combined with the TV viewing time. Compared with the group with TV viewing ≤ 1 h/day, the group with TV viewing ≥ 4 h/day was correlated with a higher risk of AD at intermediate AD-GRS (HR = 1.34, 95% CI: 1.03–1.75, *P* < 0.001) and high AD-GRS (HR = 2.18, 95% CI: 1.65–2.87, *P* < 0.001). While no correlation was observed between computer use time and AD risk in different AD-GRS groups (Fig. 3B).

### The association between screen-based sedentary activities and brain structure

**Fig. 4:**
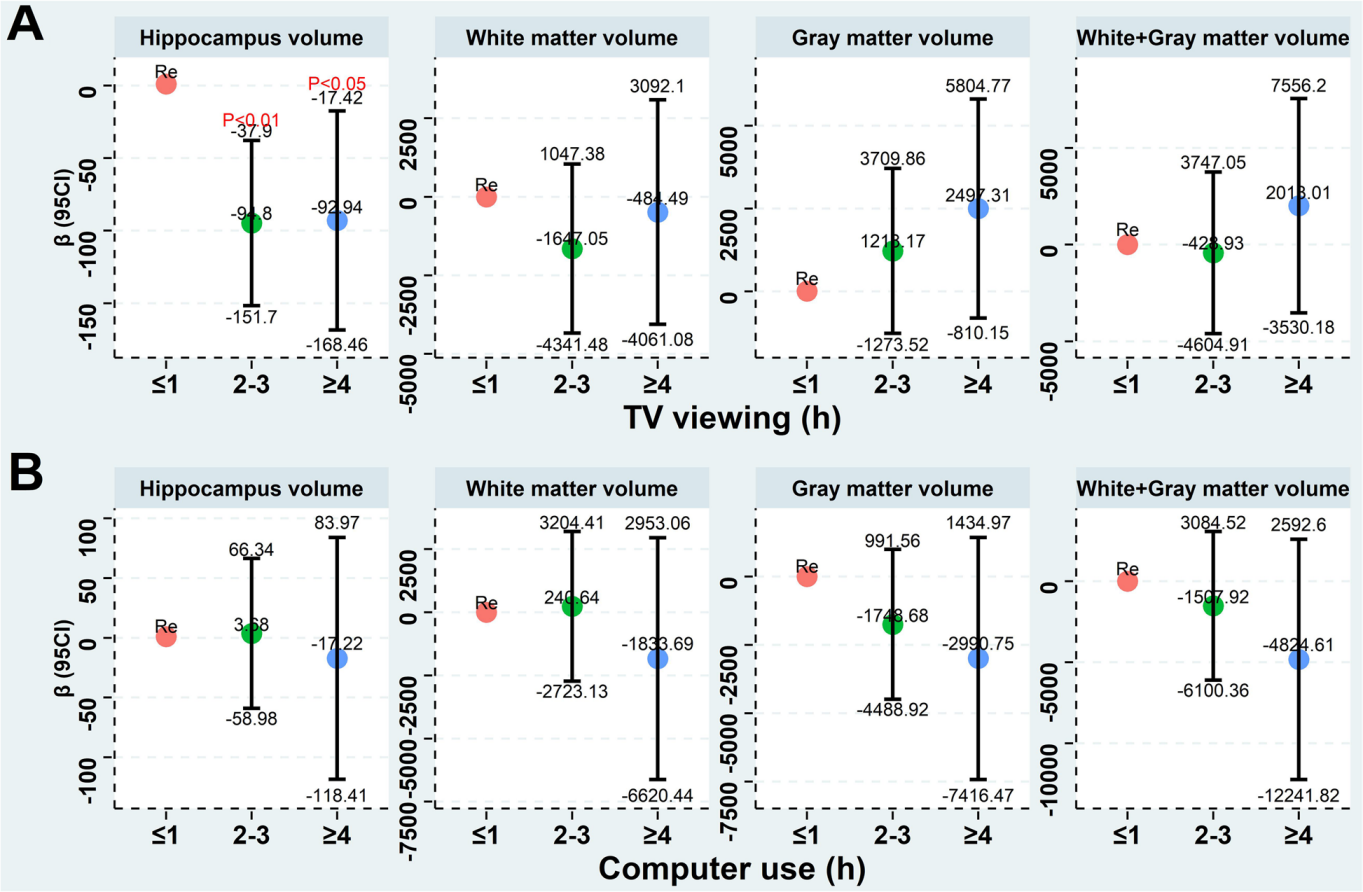
The GLM were applied to investigate the correlation between screen-based sedentary activities (Fig. 4A, TV viewing; Fig. 4B, Computer use) and gray matter volume, white matter volume, white matter + gray matter volume, and hippocampus volume. The model was adjusted for ethnicity, sex, education level, age, alcohol use, BMI, TDI, smoking, MI, diabetes, stroke, and hypertension

The GLM were used for evaluation of the correlation between screen-based sedentary activities and brain structure. As shown in Fig. 4A, after the model has been adjusted for multiple variables, no significant correlation was observed between TV viewing and gray matter volume, white matter volume, and white matter + gray matter volume (*P* > 0.05). However, compared to the TV viewing ≤ 1 h/day, a less hippocampus volume was observed in the groups with TV viewing of 2–3 h/day [β = (-94.8), 95% CI: (-37.9) -(-151.87), *P* < 0.01] and TV viewing ≥ 4 h/day [β = (-92.94), 95% CI: (-17.42) -(-168.46), *P* < 0.05]. As shown in Fig. 4B, no significant correlation was observed between computer use and brain structure.

### Summary of the MR analysis

The bidirectional MR was utilized to validate the causal association between AD and TV viewing.

**Fig. 5:**
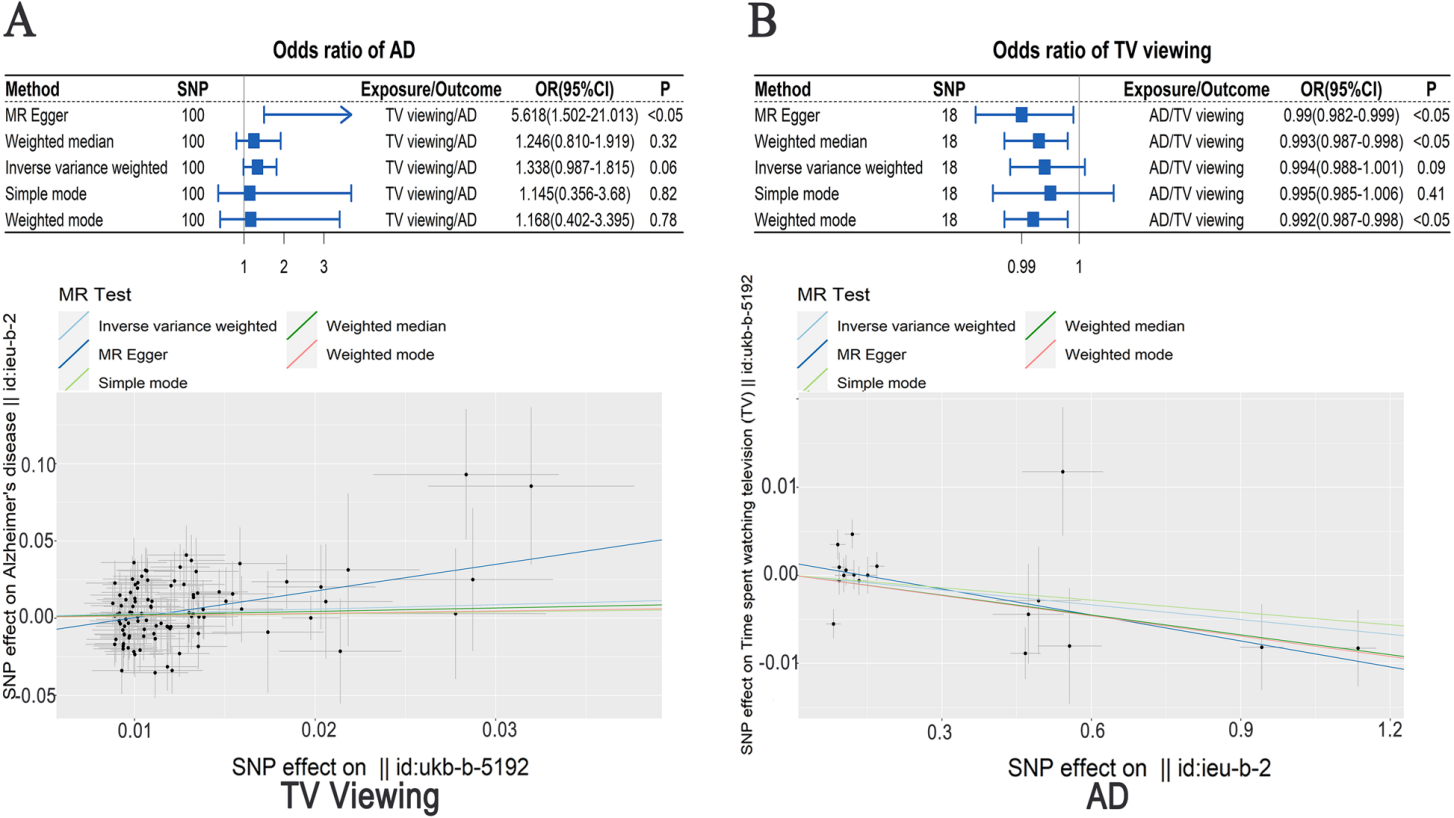
**A**: Mendelian Randomization-Based Analysis of TV viewing (exposure) and AD risk (outcome). **B**: Mendelian Randomization-Based Analysis of AD (exposure) and TV viewing (outcome)

We first investigated when TV viewing was used as an exposure factor, whether there was a causal association with AD (outcome) risk. The results of five MR analysis methods are shown in Fig. 5A. No obvious causal association was observed between TV viewing time and AD risk analyzed using Inverse variance weighted (OR = 1.338, 95%CI: 0.987–1.815, *P* = 0.06), Weighted mode (OR = 1.168, 95%CI:0.402–3.395, *P* = 0.78), Weighted median (OR = 1.246, 95%CI: 0.81–1.919, *P* = 0.32), and Simple mode (OR = 1.145, 95%CI: 0.356–3.68, *P* = 0.82), however, significant causal effect of TV viewing time was observed on AD risk analyzed using MR Egger (OR = 5.618, 95%CI: 1.502–21.013, *P* < 0.05). We further performed leave-one-out sensitivity test, pleiotropy test, and cohort heterogeneity test, and no heterogeneity was observed among IVs (*P* > 0.05), and no significant difference was observed between the MR results estimated by other IVs and the total results after excluding a certain IV, while there was horizontal pleiotropy among multiple IVs (*P* < 0.05) (Supplemental Figure S1). Visualizations of other MR results are shown in Supplemental Figure S2-S3. MR Egger regression estimation is preferred in the case with existence of pleiotropy, so using MR Egger, a significant causal association of TV viewing time with AD risk was observed, and more TV viewing time exposure was associated with a higher AD risk.

We next investigated when AD was used as an exposure factor, whether there was a causal association with TV viewing time (outcome). The results of five MR analysis methods are shown in Fig. 5B**.** Though no significant causal effect of AD was observed on TV viewing time using Inverse variance weighted (OR = 0.994, 95%CI: 0.988–1.001, *P* = 0.09) and Simple mode (OR = 0.995, 95%CI: 0.985–1.006, *P* = 0.41), we found a significant causal effect of AD on TV viewing time using Weighted mode (OR = 0.992, 95%CI: 0.987–0.998, *P* < 0.05), Weighted median (OR = 0.993, 95%CI: 0.987–0.998, *P* < 0.05), and MR Egger (OR = 0.99, 95%CI: 0.982–0.999, *P* < 0.05). We further performed leave-one-out sensitivity test, pleiotropy test, and cohort heterogeneity test, no horizontal pleiotropy was observed among multiple IVs (*P* > 0.05), and no significant difference between the MR results estimated by other IVs and the total results after excluding a certain IV, while a heterogeneity was observed among IVs (*P* < 0.01), (Supplemental Figure S4). Visualizations of other MR results are shown in Supplemental Figure S5-S6. When there is only heterogeneity and no pleiotropy, the Weighted Median results are preferred, so a significant causal effect of AD was observed on TV viewing time using Weighted Median, and more AD exposure was associated with less TV viewing time.

There were bidirectional causal effects between TV viewing time and AD as analyzed using bidirectional MR, and more TV viewing time exposure was correlated with higher AD risk and more AD exposure was correlated with less TV viewing time.

## Discussion

The findings of this study include: 1) TV viewing ≥ 4 h/day was correlated with a higher risk of VD, AD and all-cause dementia compared to TV viewing ≤ 1 h/day; 2) TV viewing ≥ 4 h/day was correlated with a higher AD risk at intermediate and high AD-GRS compared to TV viewing ≤ 1 h/day; 3) compared to TV viewing ≤ 1 h/day, TV viewing 2–3 h/day and TV viewing ≥ 4 h/day were correlated with a less hippocampus volume; 4) there were bidirectional causal effects between TV viewing time and AD analyzed by bidirectional MR; more TV viewing time exposure was correlated with a higher AD risk and more AD exposure was correlated with less TV viewing time.

Previous studies found that more watching TV time was associated with cognitive decline [8, 9, 34]. Consistently, in our large population study, TV viewing ≥ 4 h/day was correlated with a higher risk of VD, all-cause dementia, and AD. AD development has been demonstrated to be attributed to combination of environmental and genetic factors [35], and individualized interventions are critical for participants with different genetic risks [6, 36]. For the first time, our study investigated the relationship between TV viewing and AD-GRS as well as AD development, and we observed an increased HR values with higher genetic risk and prolonged TV viewing time. Therefore, it was recommended that people with intermediate and high AD-GRS should control their TV viewing time to be less than 4 h/day or even less than 1 h/day. In addition, hippocampal atrophy is one of the imaging manifestations closely related to AD [37]. In our study, TV viewing ≥ 4 h/day and 2–3 h/day were correlated with less hippocampus volume, which further provides evidence for the correlation between TV viewing time and AD risk.

Current observational studies are limited to the problem of confounding and reverse causality. Mendelian randomization (MR) is a novel analytical method for studying causal associations [38]. This study, for the first time, investigated the causal association between TV viewing and AD utilizing bidirectional MR, and we found bidirectional causal effects between TV viewing time and AD: more TV viewing time exposure was correlated with higher AD risk, and more AD exposure was correlated with less TV viewing time.

The association of TV viewing time exposure with higher AD risk can be explained as follows: first, TV viewing time is correlated with a higher risk of cardiovascular and cardiometabolic diseases [39], both of which are risk factors for AD [40]; second, watching TV increases time of sitting, resulting in reduced muscle activity and energy expenditure [41, 42], further leading to physical activity reduction and cognitive decline [43, 44]; third, TV viewing usually implies a long period of sitting after dinner in the evening, which may be harmful to cardiometabolic health [42], thereby impacting brain health [45]; forth, more TV viewing can cause passive intense sensory stimulation of the audience, and affect their emotions, so it may lead to spiritual and psychological influence [46]. In addition to its memory functions, hippocampus has been found to function in emotional coding [47]. In our study, we also found that TV viewing ≥ 4 h/day and 2–3 h/day were correlated with less hippocampus volume. In our study, after adjusting for multiple cardiovascular confounders, the correlation between more viewing time and a higher AD risk suggesting the existence of more underlying mechanisms between them, which need further study in the future.

The association of more AD exposure with less TV viewing time may be explained as follows: in AD or preclinical AD patients, a variety of clinical symptoms appear including early clinical symptoms of mood change, anxiety, disturbed sleep, apathy, and depressive symptoms, and late clinical symptoms of impaired judgment, disorientation, loss of interest and other mental behavioral changes [48, 49]. Therefore, reduced TV viewing time may be an early manifestation (loss of interest) of AD. Our bidirectional MR study provided the causal effects between more AD exposure and less TV viewing time. More underlying mechanisms need further study in the future.

In addition, our study found that there was no obvious association between computer use and dementia. On the one hand, long-term computer use may lead to a lack of exercise and physical activity and a reduction in social interaction [50], which may be associated with an increased risk of dementia [51]. On the other hand, computer use may require a certain amount of cognitive and mental activity, which helps to keep the brain active and flexible, thus reducing the risk of developing dementia [52]. More evidence is needed to confirm the link between computer use and dementia risk.

The main strengths of our study include follows: first, this study was based on the data from a prospective cohort study of UKB with large-scale long-term follow-up; second, in this study, multiple confounders associated with dementia were adjusted; third, the correlations between TV viewing, genetic risk and AD risk were studied for the first time in this study; fourth, in this study, the correlation between TV viewing and brain structural volume was studied, which provides more evidence support for the correlation between TV viewing and AD risk; finally, to avoid the influence of confounding factors, a novel causal association study method of bidirectional MR was used for the first time to investigate the causal relationship of TV viewing and AD. On the other hand, there are still some limitations in our study: first, due to limited information of other types of sedentary behaviors, we only included two popular forms of screen-based sedentary activity; second, some participants may had changed their computer use time or TV viewing time during follow-up, and lack of this information is also a limitation of our study; finally, this study mainly involved people from Europe, so the conclusions drawn have certain limitations for people in other regions, however, they can still be used as a reference for people in other regions in dementia prevention.

## Conclusions

There was a causal effect between TV viewing time and AD using bidirectional MR, and more TV viewing time exposure was correlated with a higher AD risk. Therefore, it is recommended that people with intermediate and high AD-GRS should control their TV viewing time to be less than 4 h/ day or even less than 1 h/day.

### Supplementary Information

**Additional file 1: Table S1**. The single nucleotide polymorphisms (SNPs) that showed significant genome-wide association with AD, (the SNP loci of newly discovered genes in the UKB database were not included). **Table S2.** The time spent watching television (TV) instrumental Variables (IVs) were obtained from a GWAS of UKB participants (https://gwas.mrcieu.ac.uk/; GWAS ID: ukb-b-5192). **Table S3.** Extract the instrumental Variables (IVs) information that exposure (the time spent watching television ) to the outcome(Alzheimer's disease). **Table S4.** The exposure(the time spent watching television ) instrumental Variables (IVs) were matched to the effect allele of outcome(Alzheimer's disease) instrumental Variables (IVs). **Table S5.** Alzheimer's disease instrumental Variables (IVs) were obtained from a GWAS of Alzheimer Disease Genetics Consortium (ADGC), European Alzheimer's Disease Initiative (EADI)]. (https://gwas.mrcieu.ac.uk/; GWAS ID:ieu-b-2). **Table S6.** Extract the instrumental Variables (IVs) information that exposure (Alzheimer's disease) to the outcome(the time spent watching television). **Table S7.** The exposure(Alzheimer's disease) instrumental Variables (IVs) were matched to the effect allele of outcome(the time spent watching television ) instrumental Variables (IVs)

**Additional file 2: Figure S1.** Leave-one-out sensitivity test of MR results when the time spent watching television is the exposure, AD is the outcome. **Figure S2.** Association of a single exposed SNP with outcome when the time spent watching television is the exposure, AD is the outcome. **Figure S3.** Distribution of single SNP when the time spent watching television is the exposure, AD is the outcome. **Figure S4.** Leave-one-out sensitivity test of MR results when AD is the exposure, the time spent watching television is the outcome. **Figure S5.** Association of a single exposed SNP with outcome when AD is the exposure, the time spent watching television is the outcome. **Figure S6. **Distribution of single SNP when AD is the exposure, the time spent watching television is the outcome. **Figure S7.** The Cox proportional risk models were applied to evaluate the HR of VD, AD, and all cause dementia by sex subgroup analysis. The Model was adjusted for education level, ethnicity, age, alcohol use, BMI, TDI, smoking, MI, diabetes, stroke, and hypertension. AD, Alzheimer's disease; VD, vascular dementia; TDI, Townsend deprivation index; MI, myocardial infarction; MI, body mass index; RE, reference

## Data Availability

The data are available from the UK Biobank (https://biobank.ctsu.ox.ac.uk/), but the use of these data is subject to the UK Biobank's rigorous approval process and therefore they are not publicly available.

## Abbreviations

VD: Vascular dementia
AD: Alzheimer's disease
MRI: Magnetic resonance imaging
MR: Mendelian randomization
AD-GRS: Alzheimer's disease genetic risk score
UKB: UK Biobank
TDI: Townsend deprivation index
MI: Myocardial infarction
PRS: Polygenic risk scores
SNPs: Single nucleotide polymorphisms
GWAS: Genome-wide association study
TV: Television
IVW: Inverse variance weighted
GLM: Generalized linear models
K-M curves: Kaplan–Meier curve
HR: Hazard ratio
