# Supplementary material for: Associations of screen-based sedentary activities with all cause dementia, Alzheimer’s disease, vascular dementia: a longitudinal study based on 462,524 participants from the UK Biobank
Source: BMC Public Health. 2023 Nov 2;23:2141. doi: 10.1186/s12889-023-17050-3 (PMC10621115; doi:10.1186/s12889-023-17050-3)

**Figure S1.** Leave-one-out sensitivity test of MR results when the time spent watching television is the exposure, AD is the outcome.


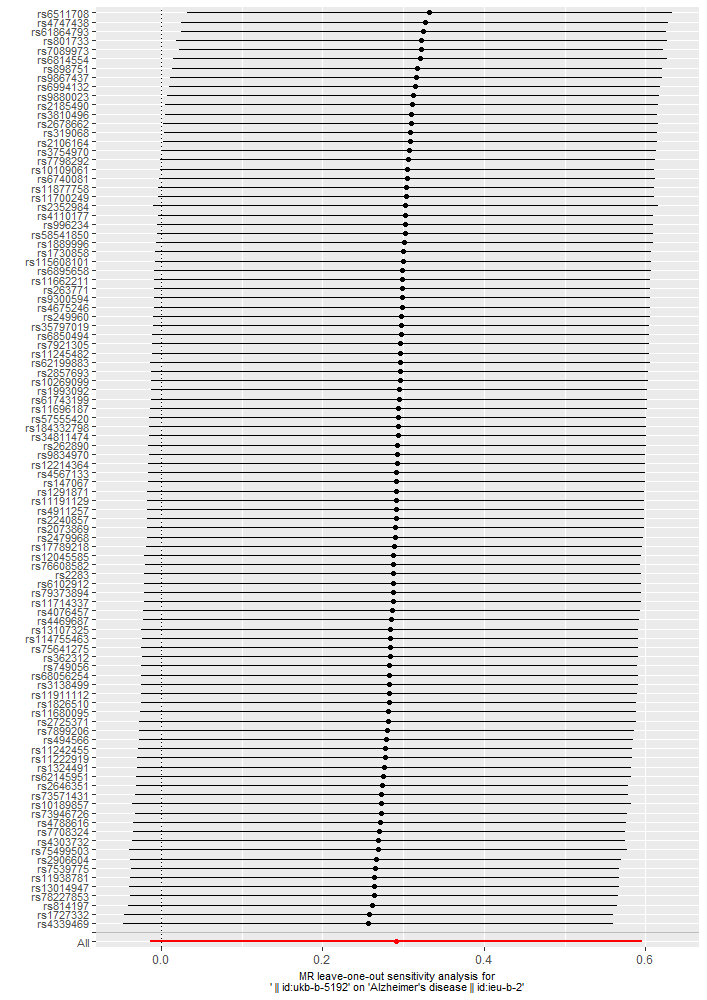


**Figure S2.** Association of a single exposed SNP with outcome when the time spent watching television is the exposure, AD is the outcome.


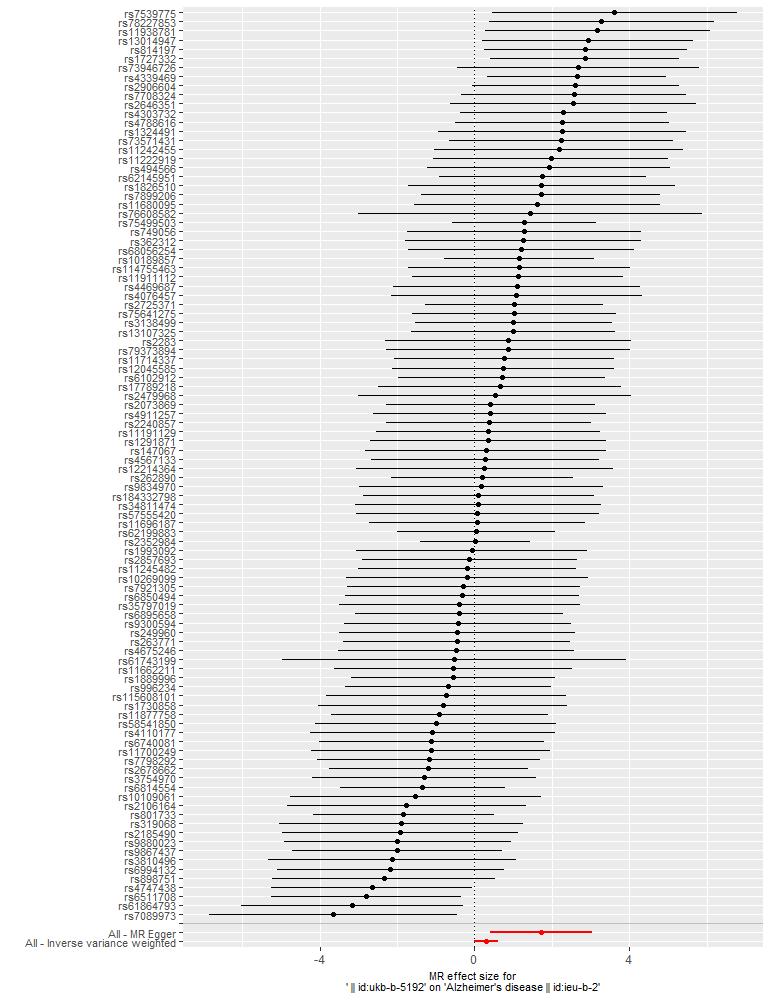


**Figure S3.** Distribution of single SNP when the time spent watching television is the exposure, AD is the outcome.


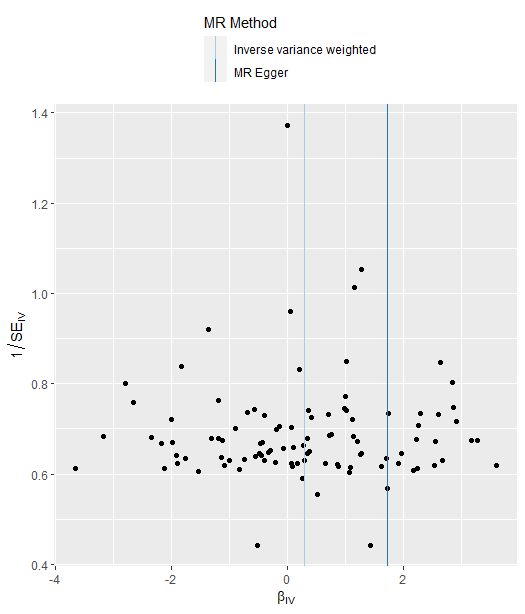


**Figure S4.** Leave-one-out sensitivity test of MR results when AD is the exposure, the time spent watching television is the outcome.


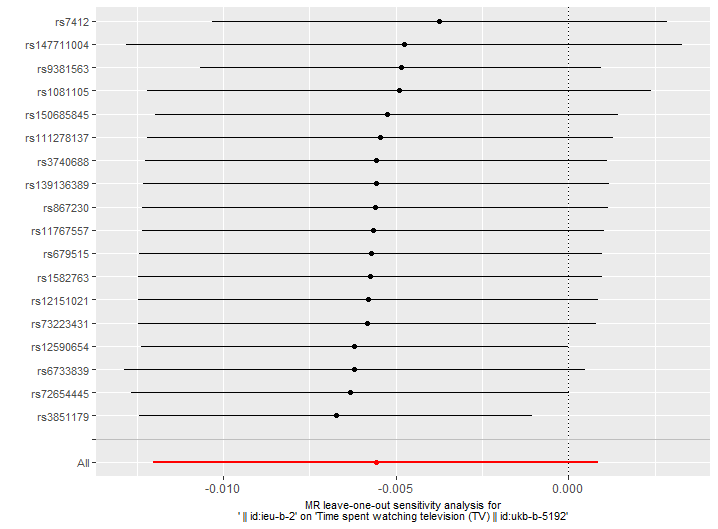


**Figure S5.** Association of a single exposed SNP with outcome when AD is the exposure, the time spent watching television is the outcome.


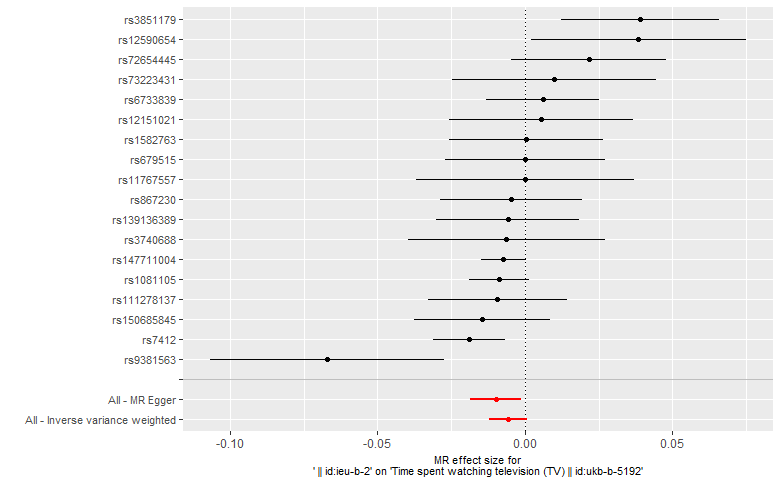


**Figure S6.** Distribution of single SNP when AD is the exposure, the time spent watching television is the outcome.


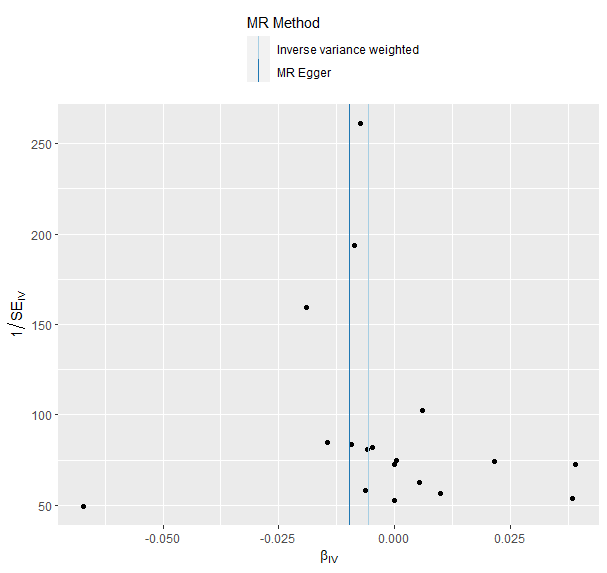


**Figure S7.** The Cox proportional risk models were applied to evaluate the HR of VD, AD, and all cause dementia by sex subgroup analysis. The Model was adjusted for education level, ethnicity, age, alcohol use, BMI, TDI, smoking, MI, diabetes, stroke, and hypertension. AD, Alzheimer's disease; VD, vascular dementia; TDI, Townsend deprivation index; MI, myocardial infarction; MI, body mass index; RE, reference.


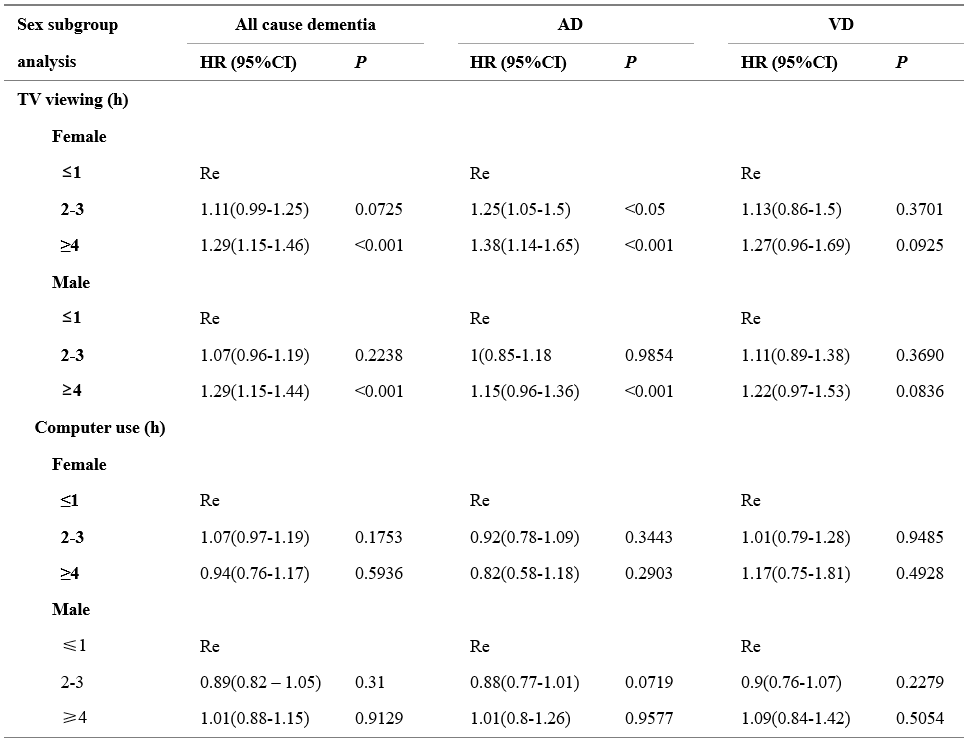

Supplement: Supplementary file 2 — Additional file 2: Figure S1. Leave-one-out sensitivity test of MR results when the time spent watching television is the exposure, AD is the outcome. Figure S2. Association of a single exposed SNP with outcome when the time spent watching television is the exposure, AD is the outcome. Figure S3. Distribution of single SNP when the time spent watching television is the exposure, AD is the outcome. Figure S4. Leave-one-out sensitivity test of MR results when AD is the exposure, the time spent watching television is the outcome. Figure S5. Association of a single exposed SNP with outcome when AD is the exposure, the time spent watching television is the outcome. Figure S6. Distribution of single SNP when AD is the exposure, the time spent watching television is the outcome. Figure S7. The Cox proportional risk models were applied to evaluate the HR of VD, AD, and all cause dementia by sex subgroup analysis. The Model was adjusted for education level, ethnicity, age, alcohol use, BMI, TDI, smoking, MI, diabetes, stroke, and hypertension. AD, Alzheimer's disease; VD, vascular dementia; TDI, Townsend deprivation index; MI, myocardial infarction; MI, body mass index; RE, reference [file 12889_2023_17050_MOESM2_ESM.docx]
